# Supplementary material for: The Wdr5-H3K4me3 Epigenetic Axis Regulates Pancreatic Tumor Immunogenicity and Immune Suppression
Source: Int J Mol Sci. 2024 Aug 12;25(16):8773. doi: 10.3390/ijms25168773 (PMC11354242; doi:10.3390/ijms25168773)
Supplement: Supplementary file 1 [file ijms-25-08773-s001.zip › ijms-3070782-supplementary.pdf]

Supplemental Data

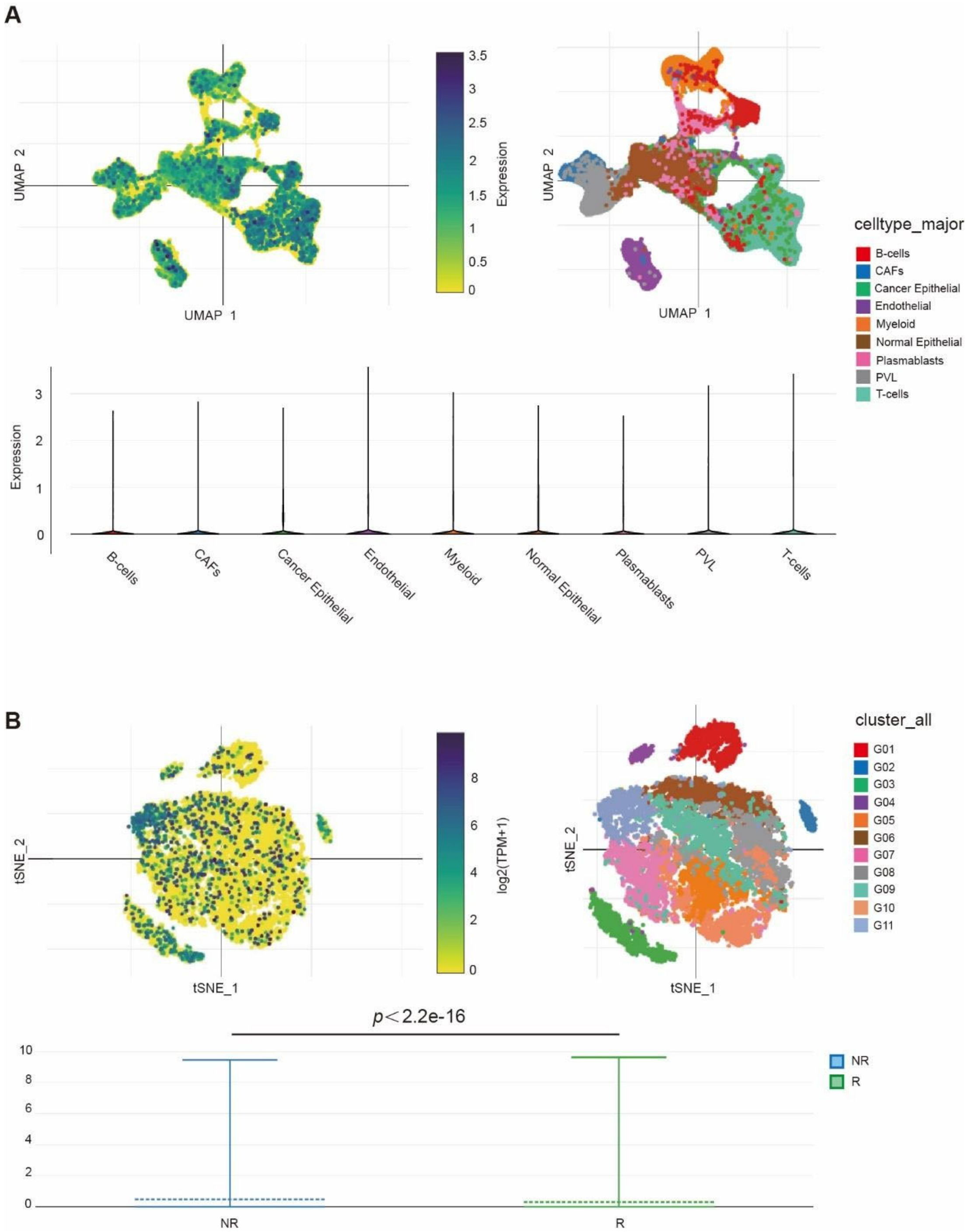

Figure S1. WDR5 expression profiles in human breast cancer and melanoma. A. UMAP of major cell subpopulations (right panel) and WDR5 expression level (left panel) in the indicated cell subpopulations in human breast cancer. The bottom panel shows violin plot of WDR5 expression level in the indicated major cell subpopulations as in the upper left panel. **B.** UMAP of cell subpopulations showing WDR5 expression level in responders and non-responders to checkpoints immunotherapy in melanoma patients.

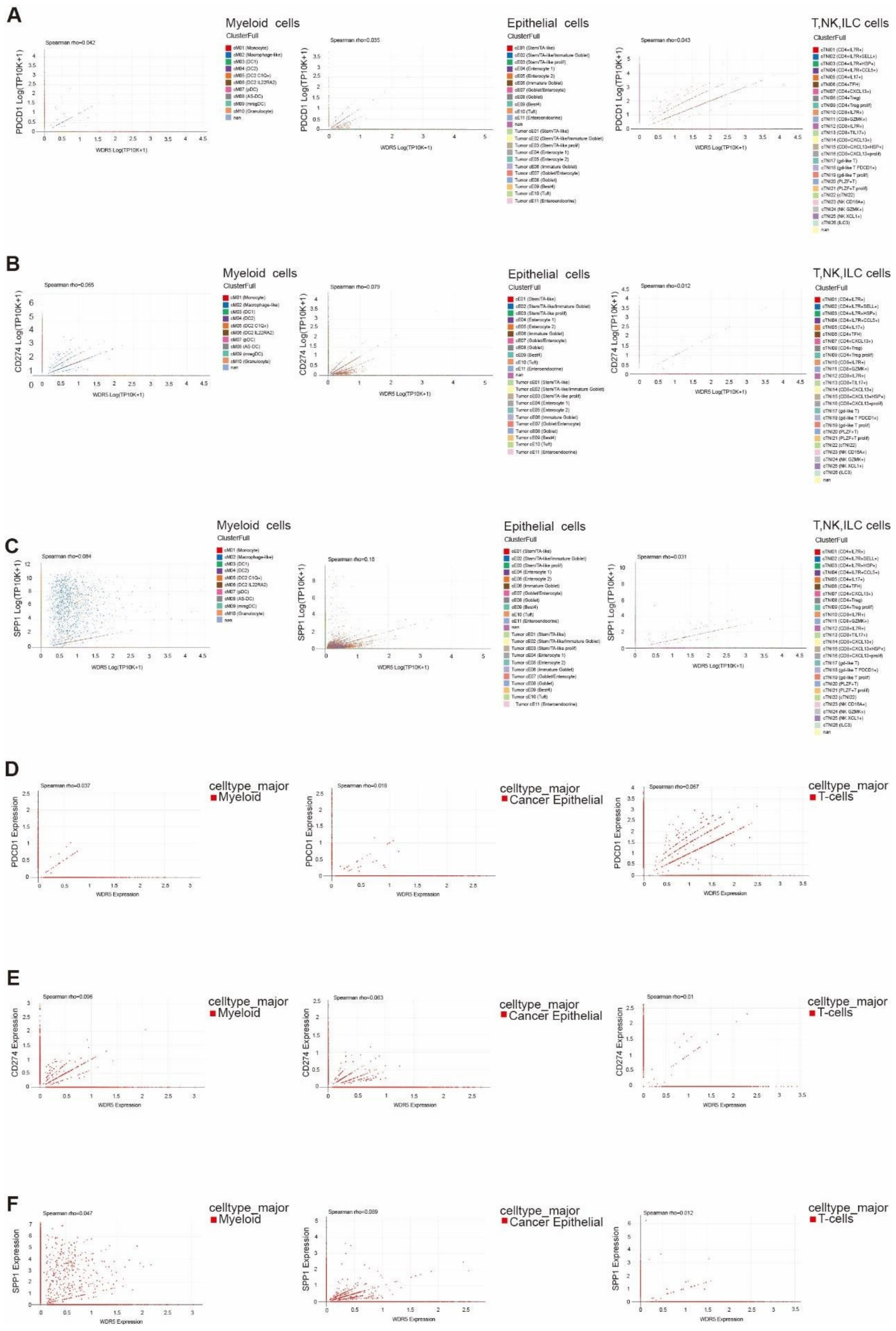

Figure S2. Correlation between WDR5 and PDCD1, CD274, SPP1 in human colon

cancer and breast cancer patients. A-C. Correlation between WDR5 and PDCD1 **(A)**, CD274 **(B)**, SPP1**(C)** in myeloid cells (left panel), epithelial cells (middle panel), T, NK and ILC cells (right panel) in colon cancer patients. **D-E.** Correlation between WDR5 and PDCD1 **(D)**, CD274 **(E)**, SPP1**(F)** in myeloid cells (left panel), epithelial cells (middle panel) and T cells (right panel) in breast cancer patients.

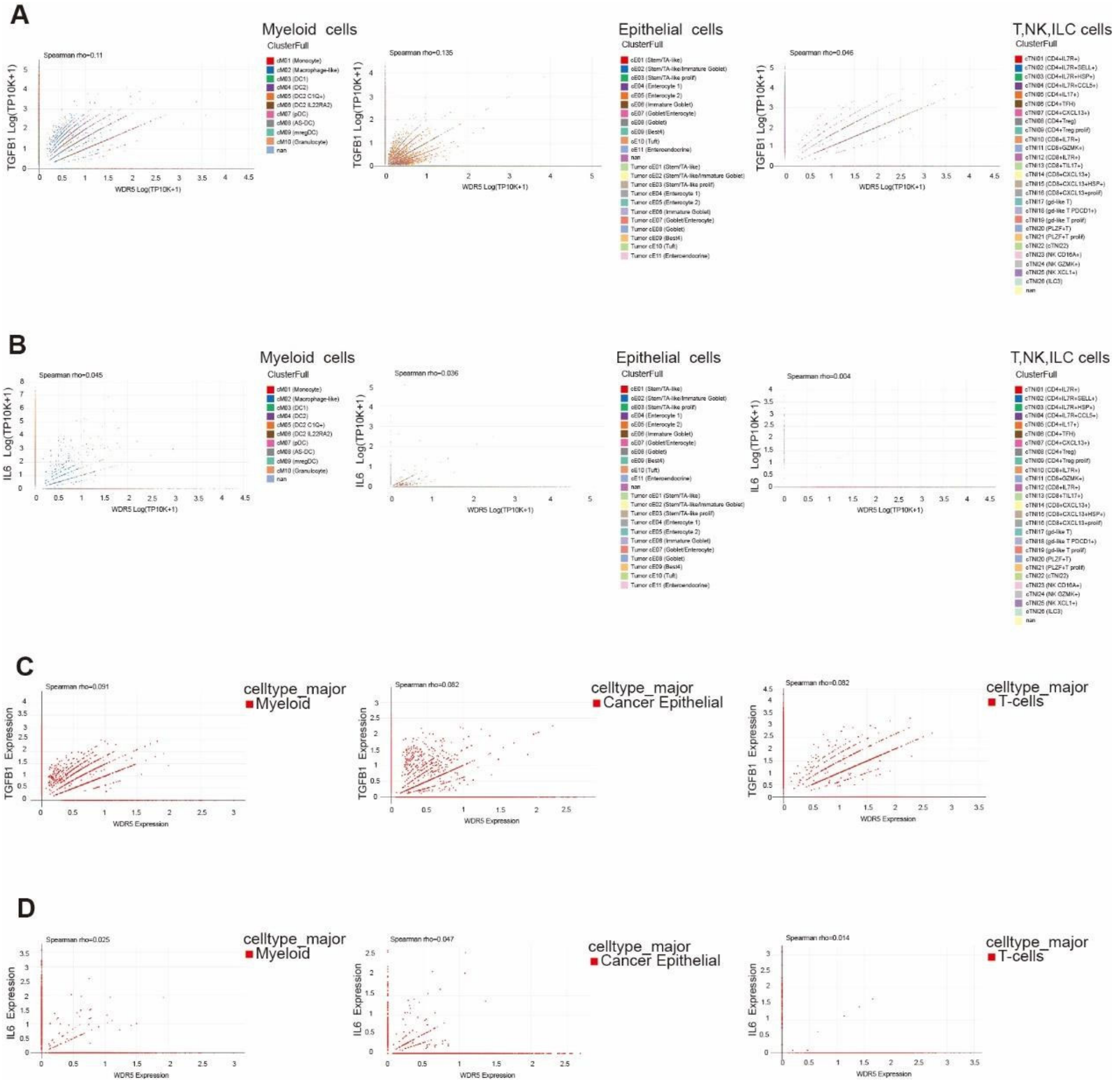

Figure S3. Correlation between WDR5 and TGFB1, IL6 in human colon cancer and breast cancer patients. A-B. Correlation between WDR5 and TGFB1 **(A)**, IL6 **(B)** in myeloid cells (left panel), epithelial cells (middle panel), T, NK and ILC cells (right panel) in colon cancer patients. **C-D.** Correlation between WDR5 and TGFB1 **(C)**, IL6 **(D)** in myeloid cells (left panel), epithelial cells (middle panel) and T cells (right panel) in breast cancer patients.

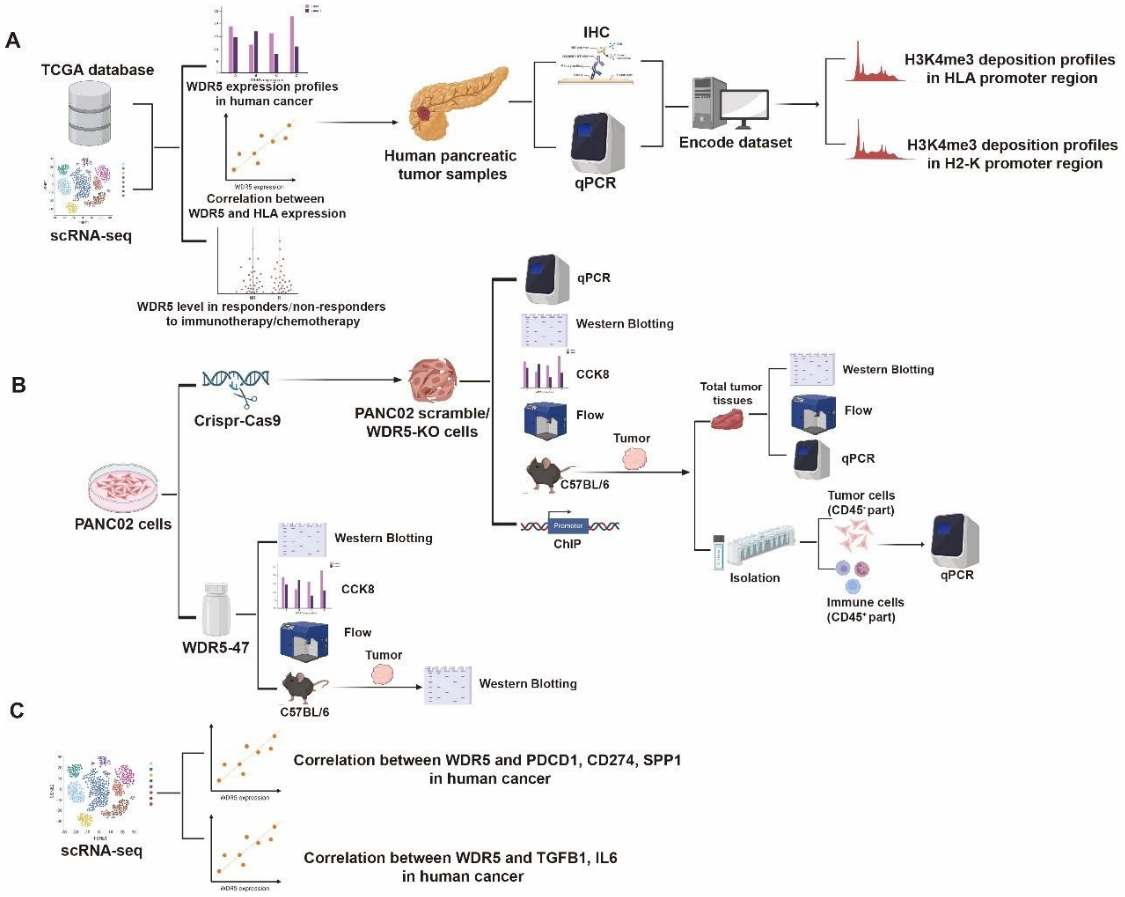

Figure S4. Chart of experimental methods. A. Experimental methods related to Fig. 1, 2 and S1. **B.** Experimental methods related to Fig. 3-7. **C.** Experimental methods related to Fig. S2 and S3.

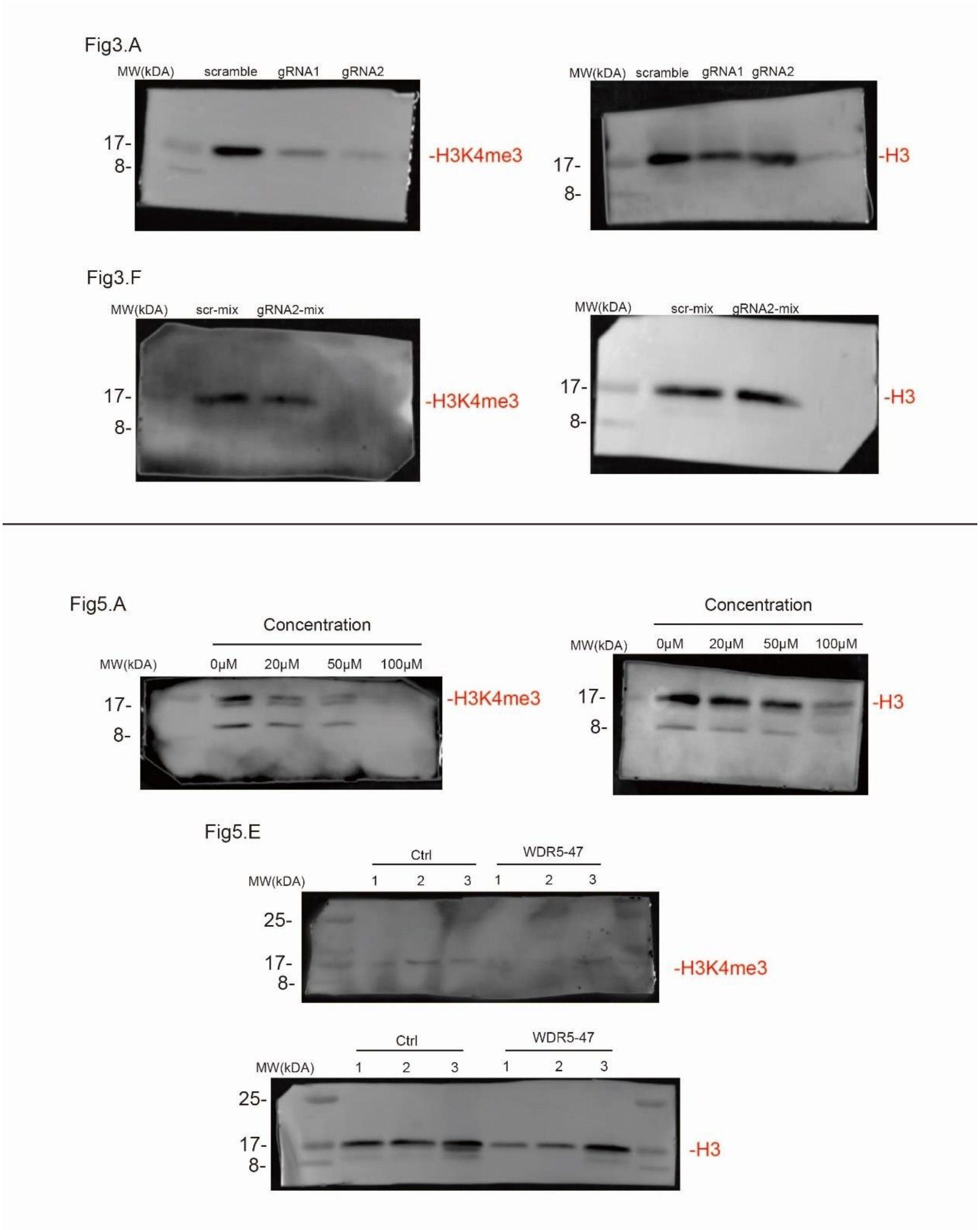

Figure S5. Original Western blots (related to Figures 3A, 3F, 5A and 5E).

Table S1. Antibodies

Name	Company	Catlog#	Application
WDR5(G-9) Mouse mAb	Santa Cruz	sc-393080	IHC
TriMethyl-Histone H3-K4 Rabbit mAb	Abclonal	A22146	WB, ChIP
Histone H3(D1H2) XP Rabbit mAb	Cell Signaling	4499	WB
FITC anti-mouse H-$2K^b$/H-$2D^b$ Antibody	BioLegend	114605	Flow Cytometry
PerCP anti-mouse CD8a Antibody	BioLegend	100732	Flow Cytometry
APC anti-mouse CD4 Antibody	BioLegend	100411	Flow Cytometry
Normal mouse IgG	Santa Cruz	sc-2025	ChIP

Table S2. Primers

Gene	Forward primer	Reverse primer
mWDR5	TTGCCAACTTCTCCGTGACA	TTCTCTAACGCTGCTGAGGC
mH2Kb	TCTTGTACCTGTCCTTCCCAGA	CTCTGCCCTTTCCTACCTGTG
mH2Db	AGCAGAGTTTCCGAGTGGAC	TTCAGGTCTTCGTTCAGGGC
mCD4	CCTCAAGATACCCCAGGTCTCG	CAAGGAAACCCAGAAAGCCG
mCD8	ACCTGGACATCAGAGCCCCTTG	AATCCTACGCTTTGCCCACC
mFas	ATGCTGTGGATCTGGGCT	TCACTCCAGACATTGTCC
mFasL	CTTGGGCTCCTCCAGGGTCAGT	TCTCCTCCATTAGCACCAGATCC
mPD-1	CCGCCTTCTGTAATGGTTTGAG	CGATTTTTGCCTTGGGGTGC
mPD-L1	ATTGCTCCTTGACTGCTGGCTG	TTCTGGGTTCCTCCTCCTTTCC
mSpp1	GCCTGTTTGGCATTGCCTCCTC	CACAGCATTCTGTGGCGCAAGG
mGZMB	GCCCACAACATCAAAGAACAGG	CCAACCAGCCACATAGCACAC
mPRF-1	CCTATGGCACGCACTTTATCACG	TTCACTGGAGACGCTGGCTTGG
mIL21	AAGAGGCAAGGGTGTAGTAAGAAGC	GGAAAGGATGTGGGAGAGGAGAC
mIFNα1	CTGAAGGACAGGAAGGACTTTGG	CTGCTGGTGGAGGTCATTGC
mIFNβ	CTGCGTTCCTGCTGTGCTTC	TCTTCTCCGTCATCTCCATAGGG
mTbX21	TGTTCCCATTCCTGTCCTTCAC	TGCTGCCTTCTGCCTTTCC
mIFNγ	CCATCAGCAACAACATAAGCGTC	TCTCTTCCCCACCCCGAATCAGCAG
mCXCL9	TCATTGCTACACTGAAGAACGGAG	ACGACGACGACTTTGGGGTG
mCXCL10	TCTCTCCATCACTCCCCTTTACC	CTTGCTTCGGCAGTTACTTTTGTC
mTGFβ	ACTGGAGTTGTACGGCAGTG	GGGGCTGATCCCGTTGATTT
mIL6	CCCCAATTTCCAATGCTCTCC	CGCACTAGGTTTGCCGAGTA
hWDR5	ACACCAAAGCAGTGTCCTCC	ATTTCCCATCATACGCGCCC
mH2K1-ChIP1	GTGTCGCCATTGTATTCCCG	TGGCGACTAAGACTTTGCCT
mH2K1-ChIP2	GGTTCGGGAACAGAACGGAC	GTCGGACCCCACTTCACAG
mH2K1-ChIP3	AGGACCGGAAGTCTCCTTACC	CCCCACAGACAGGGAGGGAT
mH2K1-ChIP4	GTTACAGTGCTCCCTCCCGC	CCTGACTCTGGGGGACAAGG
